# Continuous Measurement of Lactate Concentration in Human Subjects through Direct Electron Transfer from Enzymes to Microneedle Electrodes

**DOI:** 10.1021/acssensors.2c02780

**Published:** 2023-03-27

**Authors:** David M. E. Freeman, Damien K. Ming, Richard Wilson, Peter L. Herzog, Christopher Schulz, Alfons K. G. Felice, Yu-Chih Chen, Danny O’Hare, Alison H. Holmes, Anthony E. G. Cass

**Affiliations:** † Centre for Antimicrobial Optimisation, Imperial College London, Room 7S5, Commonwealth Building, Hammersmith Hospital Campus, Du Cane Road, London W12 0NN, U.K.; ‡ Department of Chemistry, Molecular Sciences Research Hub, Imperial College London, White City Campus, 82 Wood Lane, London W12 0BZ, U.K.; § Department of Infectious Disease, School of Medicine, St Mary’s Hospital, Imperial College London, Praed Street, London W2 1NY, U.K.; ∥ DirectSens GmbH, Muthgasse 11/2, 3. Floor, Vienna 1190, Austria; ⊥ Department of Bioengineering, Royal School of Mines, Imperial College London, Exhibition Road, London SW7 2AZ, U.K.

**Keywords:** biosensor, lactate, direct electron transfer enzyme, microneedles, continuous monitoring, in-human study

## Abstract

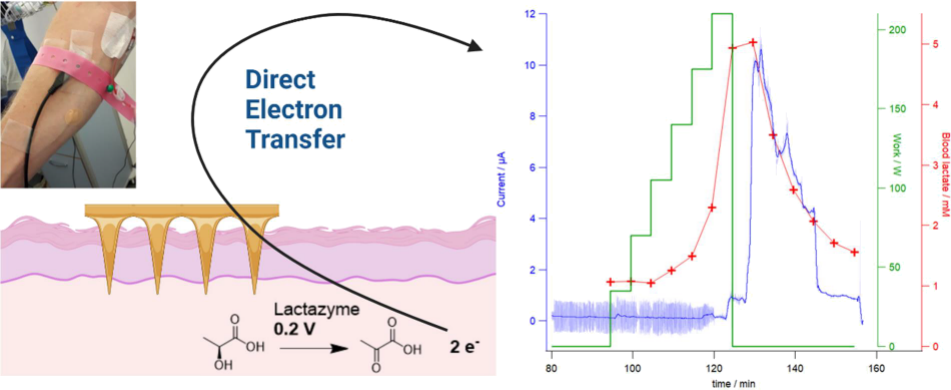

Microneedle lactate sensors may be used to continuously measure lactate concentration in the interstitial fluid in a minimally invasive and pain-free manner. First- and second-generation enzymatic sensors produce a redox-active product that is electrochemically sensed at the electrode surface. Direct electron transfer enzymes produce electrons directly as the product of enzymatic action; in this study, a direct electron transfer enzyme specific to lactate has been immobilized onto a microneedle surface to create lactate-sensing devices that function at low applied voltages (0.2 V). These devices have been validated in a small study of human volunteers; lactate concentrations were raised and lowered through physical exercise and subsequent rest. Lactazyme microneedle devices show good agreement with concurrently obtained and analyzed serum lactate levels.

Lactate concentration in blood is measured in healthcare to risk-stratify and triage patients for many different conditions. High concentrations are associated with all-cause mortality, and thus, lactate is a valuable molecule to observe as an adjunctive measurement, especially in patients at risk of deterioration.^1−5^ Measurement of lactate levels and their trends over time are directly informative in the management of sepsis, malaria, and dengue, as well as in non-infectious patient conditions including trauma and surgery.^6−8^

Blood lactate concentrations are most commonly measured through venous blood sampling and subsequent laboratory analysis. Trained staff is required to first obtain a blood sample, which must then be transported to a second location and professionally analyzed. This requires several experienced personnel and has a turnaround time of up to several hours.^9,10^ Techniques for obtaining the blood sample are necessarily invasive. Avoidance of such trauma is especially important in vulnerable populations such as neonatal and pediatric patients and may decrease risks and additional challenges associated with uncooperative patients. Lactate concentrations are therefore used primarily only in well-controlled environments, such as in hospital settings during surgery or in critical care, and are not currently in common use in community healthcare.

An easy-to-apply, inherently safe, and comfortably wearable lactate sensor would allow decision makers at all levels of pre- to post-hospital care to make better-informed choices surrounding treatment.^11^ Lactate concentrations would also be opened up as a dataset for decision makers outside critical care settings in hospitals, such as in healthcare facilities in low- to middle-income countries, carers for lower-risk patients at home, the patients themselves, and paramedics.

A microneedle array may be placed on the skin surface so that the microneedles penetrate the stratum corneum and sit in the interstitial fluid (ISF) of the viable epidermis. Concentrations of biomolecules in the ISF are linked to those in blood plasma and are especially comparable for small polar molecules such as lactate that may diffuse between the two compartments paracellularly, moving between cells rather than transcellularly through them in the case of larger molecules such as proteins.^12−15^

The microneedles themselves may be functionalized to elicit an electrical response to a change in the concentration of a target analyte, for example, by applying methylene blue modified aptamers for luteinizing hormone sensing or hydrogel enmeshed enzymes for glucose or penicillin sensing.^16−18^ Aptamers may be bound to the surface of the microneedles and can be functionalized with a redox-active molecule that will be held closer or further from the electrode surface in the presence of the analyte. Molecularly imprinted polymers are also a possible recognition element that may be grown onto a microneedle surface.^19^ The majority of published work currently uses enzymes, however.

One weakness of using enzymes as the recognition element of microneedle-based sensors is that a mediator is required to transduce an analyte-specific interaction into an electrical signal. For example, a sensor using glucose oxidase may oxidize hydrogen peroxide at the electrode surface, which is produced from the reaction of the oxidase on glucose.^20,21^ The amount of peroxide is dependent on the concentration of glucose, and therefore, the current generated from oxidizing peroxide at the anode is also dependent on the concentration of glucose. Hydrogen peroxide is oxidized at around 0.7 V relative to Ag|AgCl, which is therefore the operating voltage of a glucose microneedle sensor using glucose oxidase as the recognition element. There are however many oxidizable molecules present in the ISF, some of which are oxidized at or below 0.7 V, such as ascorbic acid and acetaminophen.^22−24^ It is desirable to create sensors that operate at lower voltages, therefore, in order to minimize off-target current generation from such molecules and thus increase overall device specificity and signal-to-noise.

Direct electron transfer (DET) enzymes are unique in their capability to interface directly with the biosensor circuit, independent of mediators. Electrons, as the product of enzymatic action on a substrate such as lactic acid, flow at a very low potential and generate a measurable current, which makes sensors more robust against electroactive interferences. Immobilizing a DET lactate enzyme within a conductive matrix on a microneedle electrode will therefore create a lactate-sensing device capable of measuring lactate concentrations within the ISF of the skin.

## Experimental Section

### Materials

The DET-type lactate enzyme lactazyme and carbon ink were obtained from DirectSens GmbH (Vienna, Austria). All other reagents were obtained from Sigma-Aldrich. Electrical wires, insulating varnish, and electrical tape were obtained from RS Components.

### Patch Fabrication

**Figure 1:**
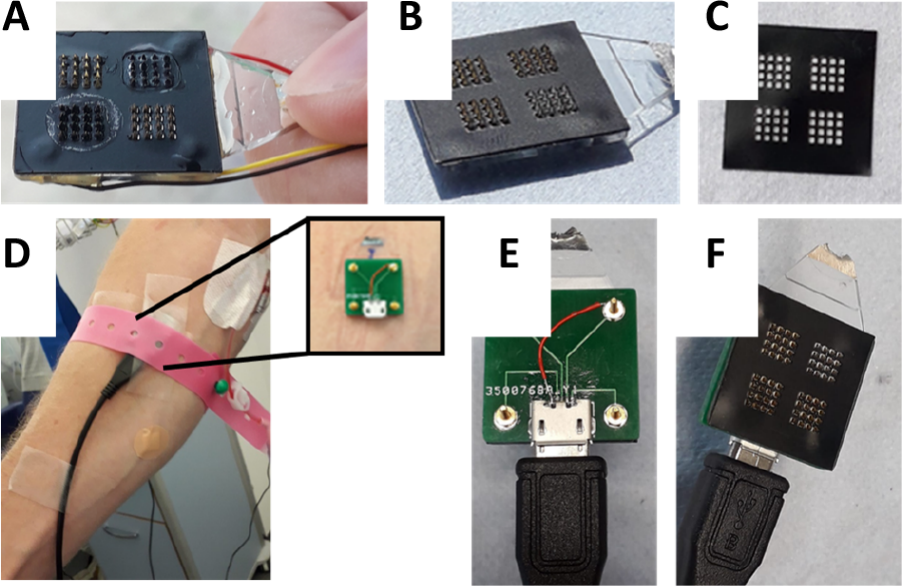
Pictures of the lactate-sensing microneedle device after (A) and before (B) functionalization, laser-cut electrical tape for insulating the base of the patch (C), the patch as used in vivo (D), and the back (E) and front (F) of the patch with a PCB attached.

Poly(carbonate) microneedle arrays were fabricated by Professor Nikolaj Gadegaard at the University of Glasgow using injection molding. Each array is 20 mm wide, and each microneedle is a square-based pyramid 1 mm in height and 0.5 × 0.5 mm at the base. The base of each needle is 0.5 mm away from its neighbor.^17^ The total active surface area of each needle is therefore 1.03 mm^2^. Each electrode (working, counter, and reference) has 16 needles, and therefore, the total active surface area of each electrode is 16.48 mm^2^. In vivo, the true area in contact with the ISF is lower and less defined, however, as the needles do not fully penetrate to their bases. The poly(carbonate) arrays were metallized at Torr Scientific (Bexhill, UK). Four separate areas of microneedles were first sputter-coated with an initial seed layer of titanium (110 nm). Following this, three sections (forming the working and counter electrodes) were coated with 150 nm of gold by e-beam evaporation, and the final section (forming the reference electrode) was coated with 150 nm of silver by e-beam evaporation. Electrical tape (PVC) was laser-cut and placed on each array so as to insulate the base of the array while leaving the microneedles uninsulated. For in vitro trials, wires were soldered to the back of the patch, and the joints were strengthened by applying the Araldite epoxy adhesive, as seen in Figure 1A. The back of the array was then insulated and made watertight with insulating varnish. For in vivo trials, a printed circuit board (PCB) was fabricated and screwed to the back of the array, which allows independent connection through a micro-USB to each of the four electrode areas, as seen in Figure 1E,F.

### Biosensor Fabrication

Biofunctionalization was performed on two designated gold working electrodes of each array, leaving a third gold electrode to act as the counter electrode and the silver electrode to act as the reference electrode after modification with sodium hypochlorite (25 μL dropcast onto the Ag electrode for 30 s and then washed with deionized water) to Ag|AgCl. Before treatment, each array was cleaned with 70% ethanol in deionized water and dried with a stream of compressed nitrogen.

Lactazyme, a recombinant enzyme derived from the previously characterized DET enzyme fcb2,^25,26^ and carbon ink solution in deionized water was dropcast (25 μL) onto each working electrode. The solution was allowed to dry at room temperature for 2 h. Cellulose acetate in acetone (3% w/v) was dropcast (25 μL) onto each working electrode. The array was allowed to dry at room temperature for 30 min. The weight percentage of cellulose acetate was varied to test the effect of the diffusion-limiting layer on the sensor’s response to lactate.

### Lactate Calibration In Vitro

The device was submerged in 10 mM phosphate-buffered saline (PBS) solution and allowed to equilibrate for 600 s at 25 °C with an applied potential of 0.2 V. The lactate concentration was modified through the addition of aliquots of 0.5 M lactic acid solution in 10 mM PBS; after each addition, the sensor was allowed to stabilize for 300 s before the next addition. The reported current for each concentration of lactate was measured as an average across 260–280 s (all data points over 20 s) after each addition, with current measured every 0.2 s.

## Results and Discussion

### Sensor Response In Vitro

Each array comprised 4 separate metallized electrode areas with 16 microneedles in each: 2 electrode areas were functionalized and acted as working electrodes, 1 area was left as unfunctionalized gold and acted as the counter electrode, and 1 area was modified as a Ag|AgCl reference electrode. The second working electrode was never required in this study; in practical terms, each sensor array was used as a standard three-electrode system (working, counter, and reference). The geometric surface area of each working electrode is 16.48 mm^2^ (each needle is 1.03 mm^2^); however, the value of the true active surface area is complicated by the three-dimensional nature of the electrodes. Diffusion will be substantially quicker to the tips of the electrode microneedles than closer to the bases, which will affect the diffusion coefficient in any quantitative calculations. In vivo, microneedles will also not be fully in contact with the ISF as the entire structure does not penetrate the stratum corneum, reducing the active surface area of the electrodes to the tips of the electrodes. It is important in this case that the surface area in contact with the ISF remains constant; practically, this means that the patch must be held securely.

Biosensors were first tested in vitro by submerging the sensor array in 10 mM PBS and adding increasing amounts of 0.5 M sodium lactate in 10 mM PBS to adjust the lactate concentration. Since DET from the heme domain enzyme to the electrode starts at around 0.1 V (see Supporting Information), a potential of 0.2 V was applied between the working electrode and the counter electrode, and the current was measured continuously (measurement taken once every 0.03 s) as the lactate concentration was modified in a stepwise fashion.

Lactate levels in healthy individuals at rest are around 1–2 mM in blood and ISF.^12,27^ During particularly intense anaerobic exercise, levels may rise to 15 mM or higher; a biosensor designed for use during exercise should therefore ideally have a linear range between 1 and 20 mM.^28,29^ These levels are subject to large changes over time, rising during exercise and recovering during rest.

A tighter range is required for sensors for use in the medical field, where patients at risk of septic shock may have lactate concentrations that are typical of healthy individuals. Patients are identified as undergoing septic shock when there is a vasopressor requirement to maintain a mean arterial pressure ≥65 mmHg and lactate >2 mM in the absence of hypovolemia.^6^ A sensor designed to be used medically will therefore need to have a linear range of around 1–5 mM and should also continually monitor concentrations over an extended period of time in order to give an early warning of rising and/or chronically high lactate levels.^1,5^

To tune the linear range of the sensors, a diffusion-limiting layer may be added. This layer slows diffusion to and from the active surface of the sensor and so extends the linear range to higher concentrations at the cost of lower observed currents and longer response times. The layer also acts to protect the active surface from mechanical stressors or in some cases biofouling.^30,31^

A cellulose acetate diffusion-limiting layer was applied to the working electrode of the lactate-sensing device. The thickness of the layer was modified by using different concentrations of cellulose acetate in an organic solvent and dropcasting onto the working electrode. Several solvents were trialed (acetone, ethyl acetate, chloroform, and toluene), and a small volume of the solution was applied. Acetone was found to cause only minimal damage to the PVC insulating layer and resulted in uniform and reproducible depositions of cellulose acetate.

**Figure 2:**
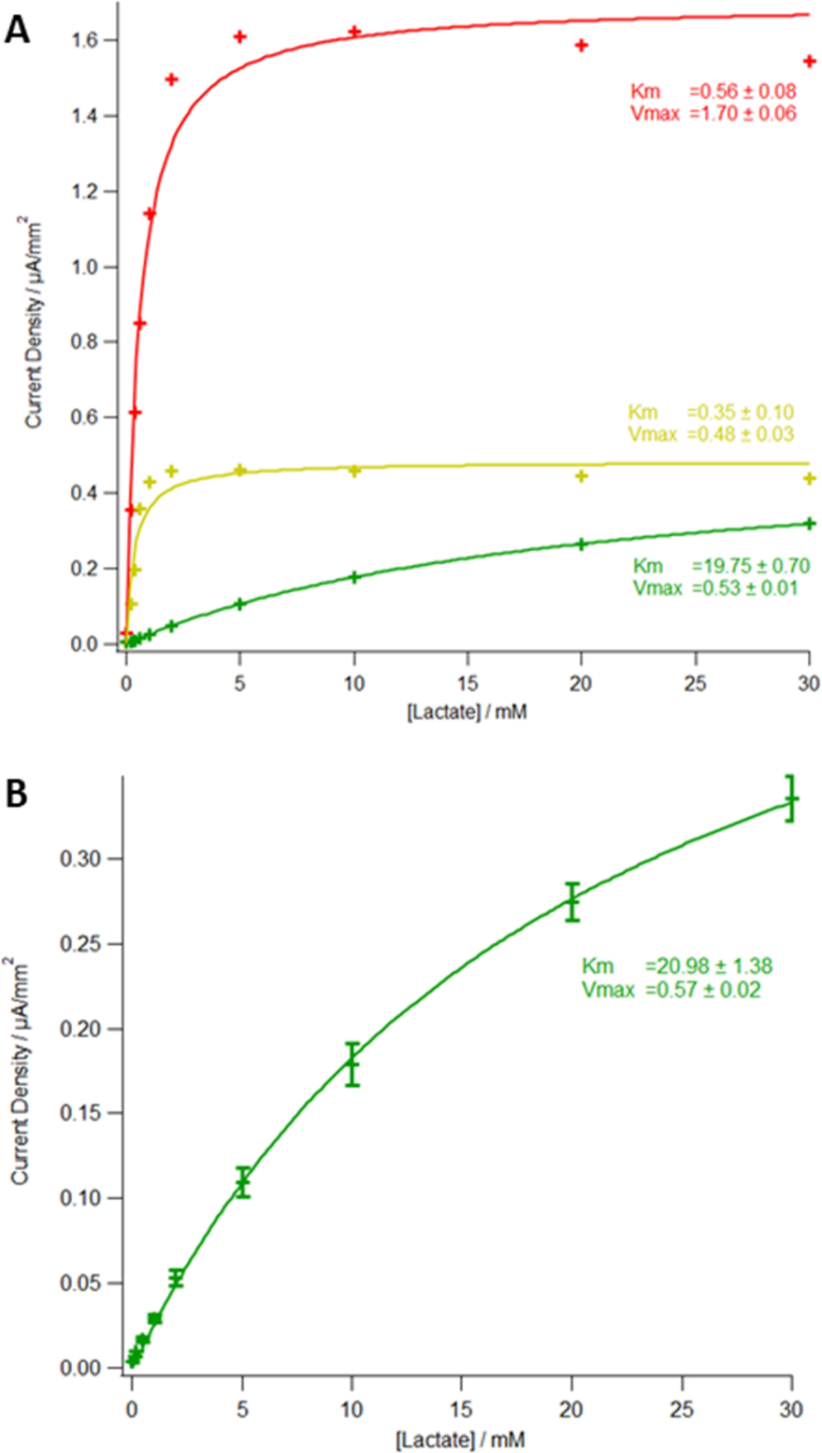
(A) Current density vs lactate concentration for devices with varying levels of cellulose acetate applied in acetone solution: 0% w/v (red), 0.3% w/v (yellow), and 3% w/v (green). (B) Current density vs lactate concentration of averaged results of three devices using 3% w/v cellulose acetate in acetone as a diffusion-limiting layer. Markers represent mean ± standard deviation (*n* = 3). All results are fitted with Michaelis–Menten equation, values of *K*_m_ and *V*_max_ are given in mM and μA/mm^2^, respectively, and the error represents 1 standard deviation.

Figure 2 shows the effect of adding a cellulose acetate diffusion-limiting layer. The largest current densities are seen without cellulose acetate (0% refers to a pure solution of acetone that was applied for the same length of time as for the solutions containing cellulose acetate); however, the sensor becomes saturated around 5 mM lactate. The addition of 0.3% cellulose acetate solution causes a decrease in the current density but does not increase the linear range appreciably. Interestingly, *K*_m_ remains similar to that observed for 0% cellulose acetate, indicating a reduction in enzymatic action (by the decrease in *V*_max_) but little or no effect on the rate of diffusion of the analyte to the enzyme. Moving to 3% cellulose acetate, however, results in a substantial increase of the dynamic range to >10 mM, albeit with approximately 5-fold reduced current densities compared to the 0% sensor.

The device was found to be stable when stored dry at room temperature for at least 4 days (Supporting Information Figure S2) and was continuously functional over the expected duration of the in vivo study of 2.5 h (Figure S3). Some loss of current was observed over the final hour of the extended runtime experiment (3.5 μA/mm^2^ decayed to 3.1 μA/mm^2^) but was judged to be acceptable to continue, especially as this seems to occur primarily at high concentrations of lactate (30 mM) that are unlikely to be reached in vivo.

### Sensor Response In Vivo

A proof-of-concept study on six individuals was performed to test the function of the continuous lactate-sensing array in vivo. The study protocol was reviewed and approved by London—Bloomsbury Research Ethics Committee (20/LO/0364) and registered on Clinicaltrials.gov on January 23, 2020 (NCT04238611). The study was sponsored by Imperial College London and conducted at the National Institute for Health and Care Research/Wellcome Trust Imperial Clinical Research Facility (Imperial College London, UK). A total of six participants were recruited (five males and one female). However, blood draws on one participant were unsuccessful after three attempts; without comparative concentrations, data were not obtained for this participant. For the five remaining participants, an array was placed on the right forearm and held in place with 3 M micropore tape and a loosely fitted tourniquet. The placement location of the sensor on the body can be any flat surface and will give a similar response unless attached to a working limb (in this case, the legs) where lactate is generated. For this study, sensor placement was kept consistent between participants on the right forearm. The array was connected to a potentiostat through a connecting wire attached to the PCB on the back of the array (distal side) and linked to the front of the array (proximal) through gold-plated screws. One functionalized electrode area of the array was monitored continuously with the counter and Ag|AgCl reference electrodes. A second functionalized electrode was not connected and was used as a redundant electrode in case the first was inoperable. The start time was adjusted for each participant to allow the sensor to stabilize before exercise was begun; typical start times were around 30–40 min after array application and polarization to 0.2 V.

**Figure 3:**
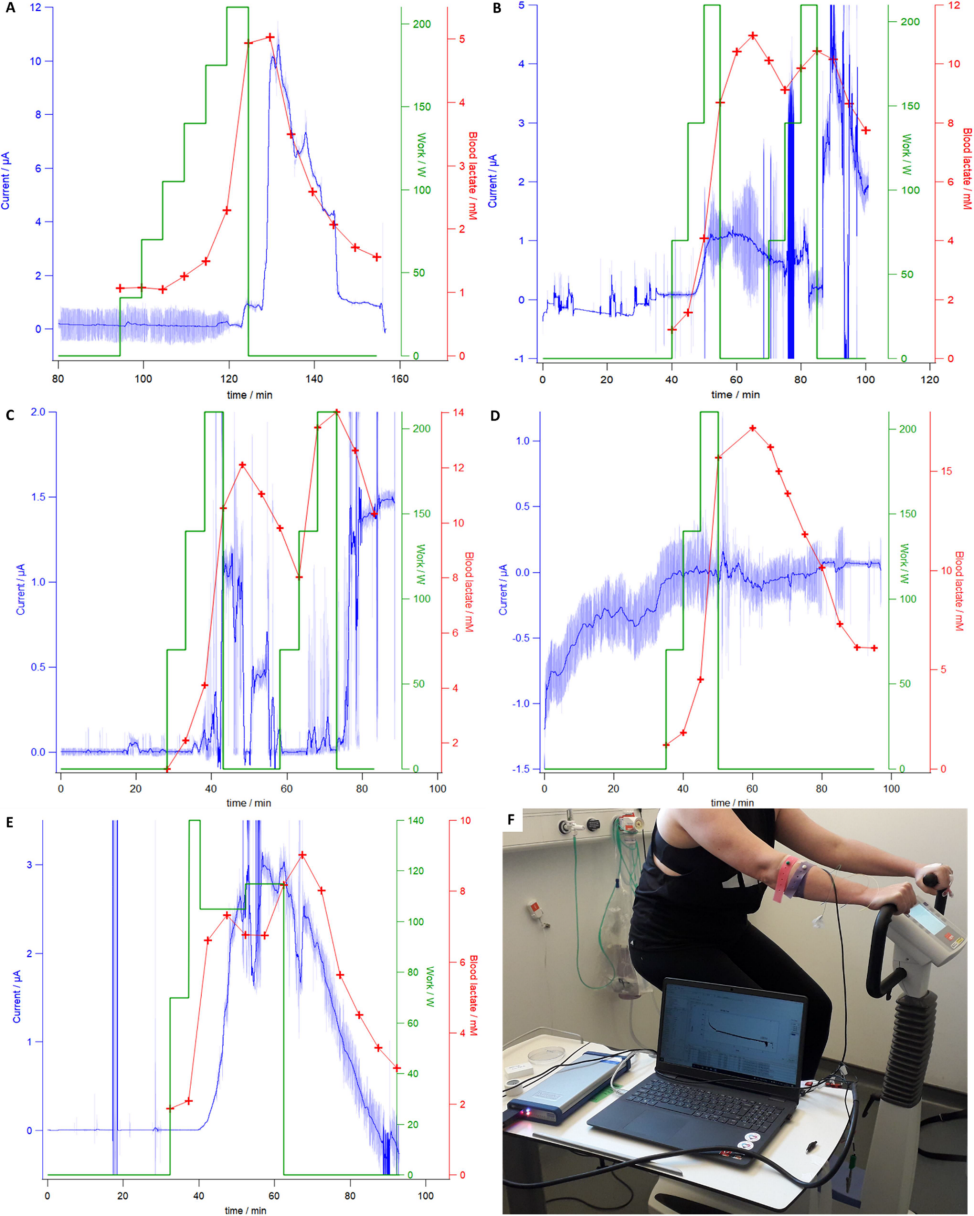
In vivo data from continuous microneedle measurement (raw data in light blue and Savitzky–Golay 2501-point second-order filtered data in dark blue) with blood lactate concentrations (red crosses) measured by a photometric lactate assay through an Architect Ci8200 from blood draws on five participants (A–E); the resistance of the bike was modulated to increase or decrease the work performed (green). Lactate levels were raised through exercise on an exercise bike (F).

Failure to secure intravenous access in one participant led to the trial being abandoned. Figure 3 shows the results of the five completed trials; the raw sensor response (data point obtained every 0.03 s) is plotted as a light blue line, and a dark blue line represents the same data with a 2501-point second-order Savitzky–Golay filter overlaid. Work performed by the participant cycling is plotted as a green line measured from the exercise bike. Venous blood lactate concentration is plotted in red. Blood samples were sent to the laboratory and analyzed using a colorimetric method: venous lactate was sampled at regular 5 min intervals and processed within 12 h at a UKAS-accredited laboratory through an Architect Ci8200 analyzer platform (Abbott, USA), beginning just before exercise started and ending after a rest period of around 30 min had ended. In trials **B** and **C**, a rest period of 10 min was introduced between two 10 min sessions of exercise in order to observe a transient rise, then fall, then rise, and then fall of lactate levels. The remaining three trials (**A**, **D**, and **E**) consisted of a single period of exercise and rest. The design of the trial was modified according to the physical condition of the participant.

**Trial A** shows a sharp increase in current at around 120–130 min, preceded by a rise in blood lactate 5–10 min before as the participant began to produce lactic acid as a result of increased athletic activity. The current then slowly falls at a similar rate as the blood lactate concentration. A sharp decrease in current is observed at 145 min; this is likely due to the patch shifting and causing a lower surface area of the microneedles to be in contact with the epidermal ISF as the participant relaxed during the recovery phase of the trial or moved from the exercise bike to a chair. The current does stabilize after this point, again following blood lactate concentration measurements.

**Trials B** and **C** introduced a rest period between two periods of exercise. This is reflected in the blood lactate concentrations for both, in which a double peak is observed. Double maxima are also shown in the current response for both trials, although neither follow the blood lactate results as well as **trial A**. Observed currents are also lower, even with slightly higher lactate concentrations, and the datasets in general are significantly noisier. **Trial C** suffers from sharp decreases to close to 0 A, implying that the array had become disconnected from the ISF. As a result of these trials, arrays in subsequent trials were more carefully taped to the participant and closely monitored to ensure that the total electrode surface area in contact with the epidermal ISF remained consistent. The array must be stably held; however, force applied to the top of the array will have the effect of forcing ISF away from the area it is placed, making the results unrepresentative of what is happening in the rest of the body, so care must be taken. The overall shape of both trials, though noisy, does follow blood lactate concentrations.

**Trial D** showed very little response, remaining at consistently low currents throughout the trial, and did not appear to track with blood lactate levels, which rose and fell as expected from previous and subsequent trials. The reasons for this failure may be tentatively attributed to either general patch application and adhesion or failure of the analyte recognition portion of the array. Electrical noise is of a similar level to other trials, which suggests that the electronic portions of the setup were functioning correctly. If the biosensor was not placed correctly or was pushed out of the stratum corneum and the epidermal ISF or if that portion of the biosensor was not responsive to lactate, then no or little change in current response would be present, as is the case for this trial. The mechanical stability of the device when held onto the skin will be an important aspect of future devices of this type.

Much like **trial A**, **trial E** shows good agreement between the blood lactate concentration and the current response of the microneedle array. Current followed blood lactate by around 5–10 min once exercise was begun, but unlike **trial A**, the current fell before, rather than after, a decrease in blood lactate was observed. Individual variations in perfusion of blood vs ISF between participants could explain this difference; the dynamics of clearing lactate from the ISF to blood are complex and variable. This trial was also in good agreement with blood lactate concentrations. Lactate generation of **trial E** was modulated dynamically based on the condition of the participant and as such had a less defined peak and decay profile. The first peak of **trial E** occurring at 45 min is followed around 5 min later by a peak in the current response of the sensor. Work was lessened at this time and then increased slightly at 50 min. A clear second peak is visible in the blood lactate at 65 min, which then decays during the rest period. The device response seems to be only a single peak, however. This may be due to the dynamics of lactate moving into and out of the ISF from the blood stream; a smoother profile may therefore be expected. It could also show that the device is not capable of picking up this small change in concentration, although calibrations in vitro and the continuous monitoring nature of the device make this explanation less likely.

The current generated from the devices follows the trend of the blood lactate results; however, the values obtained are substantially variable between trials. This means that the device is not able to give an accurate, quantitative reading of the lactate concentration of an individual’s ISF. Given the small number of volunteers and their individual variation in response to exercise (depending on gender, age, fitness, degree of sweating, and lactate threshold) as seen from the blood lactate levels, it is difficult to draw any general conclusions. There is also measurement uncertainty associated with both the sensor (as seen by the unfiltered currents) and the blood lactate values. Moreover, microdialysis studies have shown that even in the absence of exercise, skin is a significant source of lactate.^32^ These factors together make it difficult at this stage to draw more quantitative conclusions.

Trends in the overall data appear encouraging, however. While absolute values of current compared to the blood lactate concentration are variable, four of the five completed trials showed general agreement in basic rising and falling lactate concentrations. In the context of a device that may give an early warning of sepsis in at-risk patients, sensing a rise in the lactate concentration over time (as opposed to a simple concentration at a single time point) will allow for an automated early warning. As the device fabrication becomes more robust, variations due to batch-to-batch and within-batch differences will be lessened and may allow for more quantitative data to be obtained.

After glucose, lactate is the most frequently studied analyte for microneedle-based biosensors in the literature.^33^ To the best of our knowledge, all previous solid microneedle sensors use lactate oxidase (LOx) to produce hydrogen peroxide in the presence of lactate, which is measured through amperometry. The limits of detection of these sensors are often low enough (<0.1 mM) to facilitate in vivo measurements of basal lactate (1–2 mM);^12,27^ however, the sensors are often limited at higher concentrations seen in exercise or illness (10–20 mM)^28,29^ by their drop-off in the linear range, even when extended with a diffusion-limiting layer.^34−38^ An alternative to LOx is therefore attractive for enzyme-based lactate-sensing. The DET enzyme used herein required a diffusion-limiting cellulose acetate layer to extend the linear range of the sensor (similar to previous LOx-based sensors); however, it shows an enhanced linear range in vitro (Figure 2) without substantial optimization. Moreover, the enzyme functions at low applied voltages compared to LOx, which is advantageous for minimizing the effect of redox-active interferants. The lactazyme therefore represents an advantageous alternative as a lactate-specific enzyme for biosensing.

### Sensor Response to Interferants

**Figure 4:**
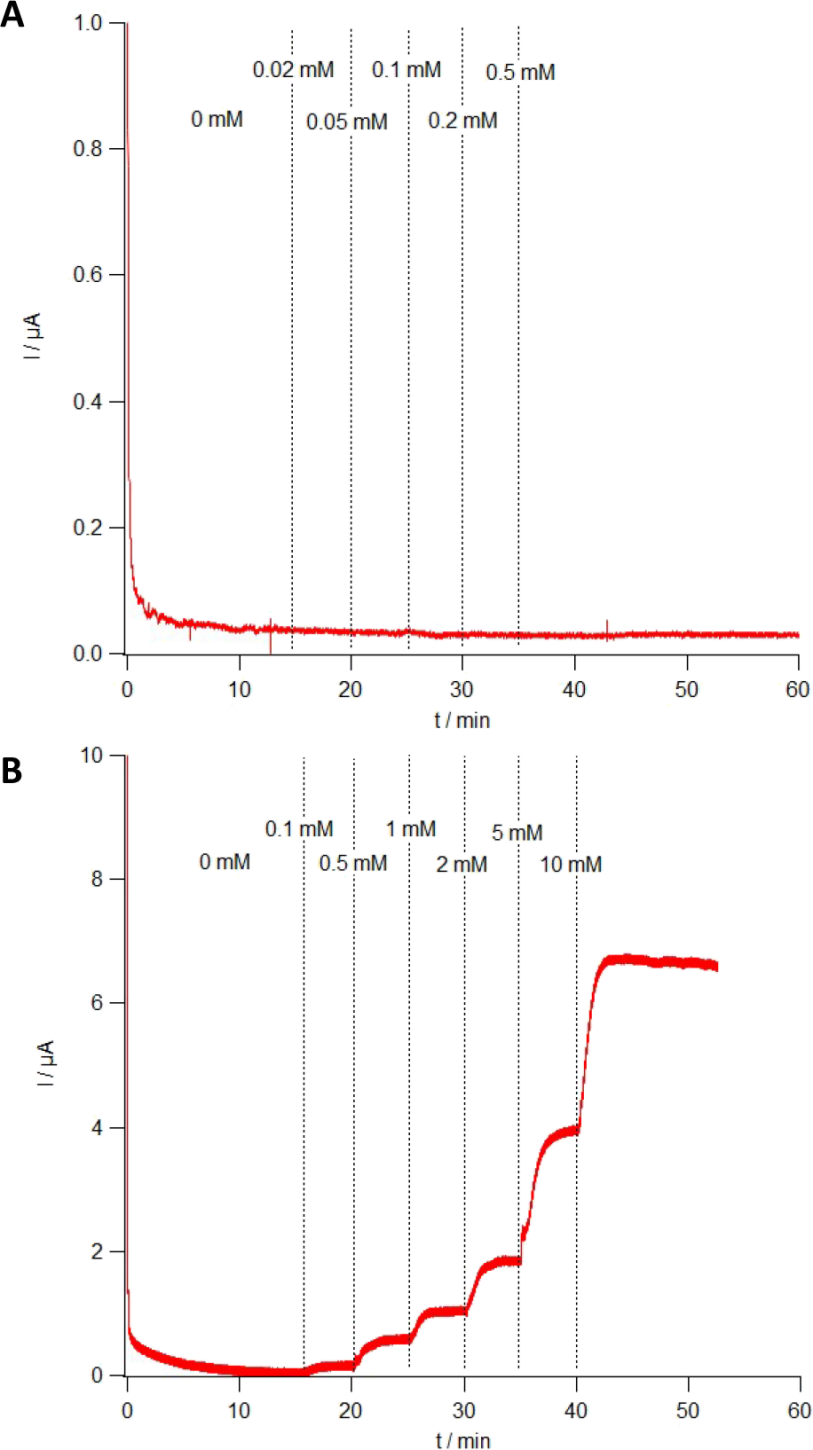
In vitro calibrations of the microneedle device against acetaminophen (A) and ascorbic acid (B) at 0.2 V. Dotted lines denote time points of the addition of stock solution to adjust the concentration to the level shown.

Sensors were also calibrated in vitro against redox-active interferants common in the ISF. Acetaminophen (typical serum concentration after oral dose <1 mM)^39^ is commonly taken to alleviate pain and so can be present in the ISF at variable and (unless measured or carefully dose-controlled) unpredictable concentrations. The low operating voltage of the sensor of 0.2 V has no effect on acetaminophen (Figure 4A), so no current will be generated in its presence. Ascorbic acid (typical serum concentration <0.1 mM)^40,41^ however is oxidized and generates current at an applied potential of 0.2 V (Figure 4B). Levels in the ISF tend to be low and change little over time; concentrations were tested at higher levels than likely to be present in the ISF to show the effect of oxidizable molecules on sensors of this type. Higher, more variable levels are seen within cells, but in the ISF, ascorbic acid will contribute to, but not overwhelm, the background noise of this sensor in its current form. While the contribution to variable background noise will be low and predictable in the case of ascorbic acid, this highlights the importance of the operating potential in the specificity of any electrochemical sensor used in vivo as other redox-active molecules may have less predictable concentration profiles. A smaller applied potential will result in fewer off-target oxidations/reductions of redox-active molecules and negates the need for additional physical filtering using, for example, a Nafion layer on the surface of the microneedles.

The largest source of variability between the sensors observed during the trials was due to initial application and mechanical stability of the device on the skin. Microneedles must be reliably, continuously, and securely held within the ISF for effective measurements to be obtained. A tight band holding the sensor to the arm is counterproductive as the ISF is forced away from the area of interest, whereas a loose band or medical tape alone in some cases results in displacement by extrusion of the microneedle tips by the inherent elasticity of the dermis. Product development of a microneedle device should take this into account when considering the method of skin attachment.

As in previous microneedle studies, pictures were taken of the attachment site after device removal to track any damage or irritation to the skin the device may have caused (see Supporting Information Figure S4).^11,17,18^ The marks left by the device faded quickly in all trials and were completely gone within 2.5 h in all participants.

## Conclusions

A lactate-sensing device prototype has been fabricated and shown to be functional in human volunteers. It runs at very low applied potentials (0.2 V), which minimizes non-specific noise from off-target oxidations. Variability between batches of devices is a concern but will be improved with the process of fabrication being more fully automated and quality management systems being implemented. Of the five in-human trials, two showed excellent agreement between the device output and measured serum lactate levels obtained from blood draws. Two other trials were in good agreement, and one gave a low, non-specific response.

The device in its current form requires extensive wiring and is impractical for routine use. Miniaturization of the electronics and the incorporation of a battery and Bluetooth (or other wireless) connectivity components are underway and will result in a device that is more easy to use. The hardware enabling such approaches is already available commercially.

Through easy-to-apply and easy-to-interpret point-of-care sensing of lactate levels, patients at risk of developing sepsis or those with conditions where sepsis heralds a negative outcome will be more able to quickly receive aid at critical junctures. In situations such as surgery where lactate levels are already monitored for this reason, a cheap, minimally invasive, and continuous monitoring device has significant advantages over laboratory testing, which takes significant time to obtain data points and is expensive in time cost of expert analysts as well as financially. Minimizing the need for blood sampling also decreases associated risks and is especially valuable when treating babies and children. A convenient and accurate lactate-sensing device provides excellent benefits in these scenarios.

## Supporting Information Available

Pictures of the device attachment site after removal of the device, graphs showing the stability of the device in storage and operation, raw chronoamperometry data over an extended period of time, and cyclic and square wave voltammograms showing characterization of the lactazyme sensor architecture for proof of DET (PDF)
