# Supplementary material for: Continuous Measurement of Lactate Concentration in Human Subjects through Direct Electron Transfer from Enzymes to Microneedle Electrodes
Source: ACS Sens. 2023 Mar 27;8(4):1639–47. doi: 10.1021/acssensors.2c02780 (PMC10152478; doi:10.1021/acssensors.2c02780)

Supporting Information: Continuous Measurement of Lactate Concentration in Human Subjects through Direct Electron Transfer from Enzyme to Microneedle Electrode

David M. E. Freeman*[1,2], Damien K. Ming[1,3], Richard Wilson[1,3], Peter L. Herzog[4], Christopher Schulz[4], Alfons K. G. Felice[4], Yu-Chih Chen[5], Danny O'Hare[1,5], Alison H. Holmes[1,3], Antony E. G. Cass[1,2].

[1]*Centre for Antimicrobial Optimisation, Imperial College London, Room 7S5, Commonwealth Building, Hammersmith Hospital Campus, Du Cane Road, London, United Kingdom, W12 0NN*

[2]*Department of Chemistry, Imperial College London, Molecular Sciences Research Hub, White City Campus, 82 Wood Lane, London, United Kingdom, W12 0BZ*

[3]*Department of Infectious Disease, Imperial College London, School of Medicine, St Mary's Hospital, Praed Street, London, United Kingdom, W2 1NY*

[4]*DirectSens GmbH, Muthgasse 11/2, 3. Floor 1190, Vienna, Austria*

[5]*Department of Bioengineering, Imperial College London, Royal School of Mines, Exhibition Road, London, SW7 2AZ*

Corresponding Author: d.freeman@imperial.ac.uk

Pages: S1-4

Figures: S1, S2, S3, S4

Figure S1CV and square wave voltammograms characterization of LactZyme sensor architecture to show proof of DET.

Measurements

- Sensors were mounted horizontally at 37°C in 50 mM PBS, pH 7.4
- CV/SWV parameters as followed were used
    - CV: 10 mV/s
    - SWV (square wave):
        - amplitude: 30 mV
        - frequency: 2 Hz
        - step potential: 5 mV

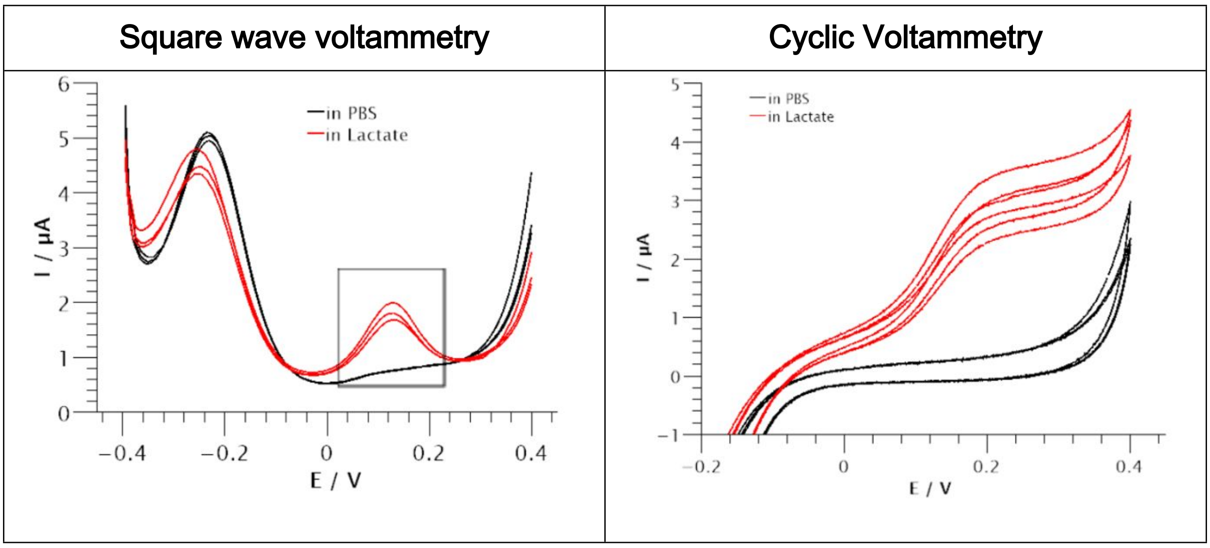

Figure S2 Calibration curves of sensors produced and tested on the same day (blue) and produced and stored dry at room temperature for 4 days then tested (purple). Points are fitted with Michalis-Menten curves, Km and Vmax are given in mM and µA respectively.

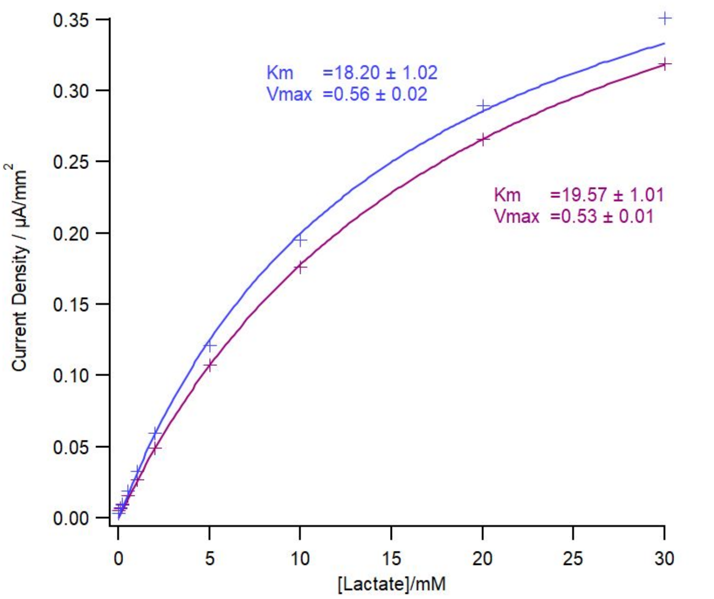

Figure S3

Raw chronoamperometry data over an extended period of time. The device was submerged in 20 mL of 10 mM PBS and a bolus of lactate solution (0.5 M in 10 mM PBS) was used to adjust the lactate concentration of the solution from 0 to 0.1, 0.2, 0.5, 1.0, 2.0, 5.0, 10.0, 20.0 and 30.0 mM at time points 0.50, 0.58, 0.67, 0.75, 0.83, 1.00, 1.17, 1.33 and 1.50 h respectively.

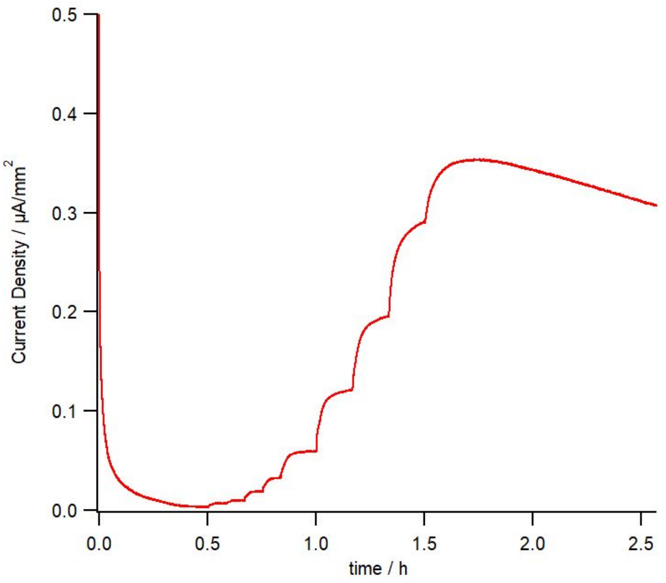

Figure S4

Pictures were taken after removal of the microneedle array at regular time intervals. While initially the marks are clear these fade quickly and in all cases were completely gone within 2.5 h.

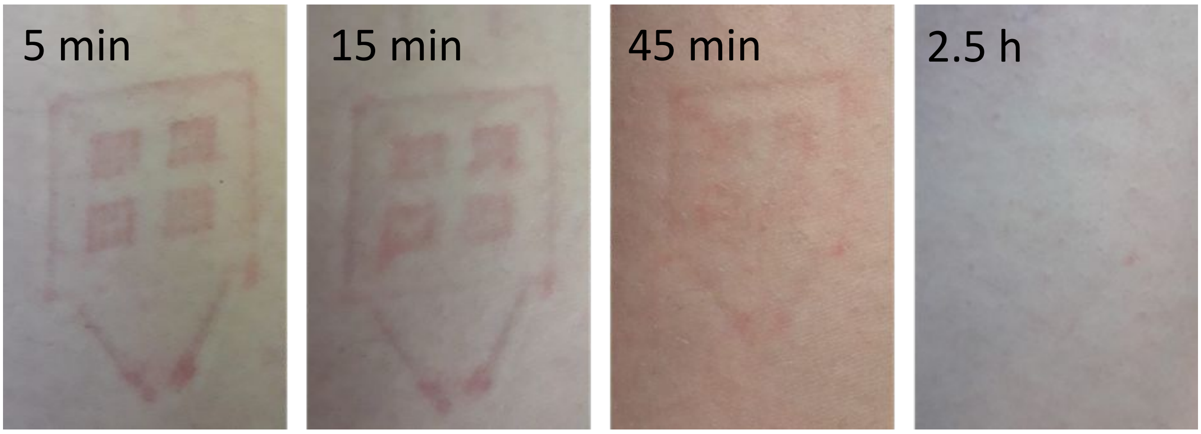

Supplement: Supplementary file 1 — se2c02780_si_001.pdf [file se2c02780_si_001.pdf]
